# Gingival phenotype distribution in young Caucasian women and men – An investigative study

**DOI:** 10.1002/cre2.482

**Published:** 2021-11-11

**Authors:** Kai R. Fischer, Jasmin Büchel, Frederic Kauffmann, Christian Heumann, Anton Friedmann, Patrick R. Schmidlin

**Affiliations:** ^1^ Clinic of Conservative & Preventive Dentistry, Division of Periodontology & Peri‐implant Diseases, Center of Dental Medicine University of Zurich Zurich Switzerland; ^2^ Department of Oral and Maxillofacial Surgery, Centre for Dental Medicine University of Freiburg Freiburg Germany; ^3^ Faculty of Mathematics, Informatics and Statistics, Department of Statistics Ludwig‐Maximilians‐University Munich Munich Germany; ^4^ Department for Periodontology, Faculty of Health Witten/Herdecke University Witten Germany

**Keywords:** gingival phenotype, phenotype distribution, probe transparency, Ramfjord teeth

## Abstract

**Objectives:**

To evaluate the relationship between gingival phenotype and tooth location based on selected index teeth (“Ramfjord”) and assess possible differences between women and men.

**Material and Methods:**

Thirty‐six women and 20 men voluntarily participated in this investigation with an average age of 23 years (min: 19; max: 37). Gingival phenotypes (GP) were assessed by transparency of a periodontal probe through the buccal gingival margin.

**Results:**

A comparable and similar GP on all index teeth was only found in seven out of the 56 subjects, that is, thin or thick only: Five participants (three male/two female) showed a uniform and constantly thick and two females a constantly thin GP. While the majority of molars (94.6%; *p* = 0.006) showed a thick GP, premolars (61.6%; *p* = 0.09) as well as incisors (70.5%; *p* = 0.046) were predominantly categorized as thin. In addition, significantly thicker GP was in general observed for maxillary teeth (*p* = 0.001) but without differences between genders (*p* = 0.722).

**Conclusion:**

No constant GP can be expected within one dentition. The use of the “Ramfjord teeth” may serve as a quick overview and reliable method to screen GP distribution.

## 1 INTRODUCTION

Historically, two different gingival biotypes were described in the literature: a thick and a thin biotype (Müller & Eger, 1997; Seibert & Lindhe, 1989; Weisgold, 1977). Based on clinical observations, the hypothesis of specific gingival and tooth properties with a thin or thick biotype was proposed (Olsson et al., 1993; Olsson & Lindhe, 1991; Weisgold, 1977). The term “biotype,” however, rather reflects a group of individuals sharing the same genotype while in the case of different gingival architectures of different thickness the term “phenotype” (GP) would probably be more appropriate. Recently, the term periodontal phenotype has been introduced by the 2017 World Workshop on the Classification of Periodontal and Peri‐Implant Diseases and Conditions to describe the combination of the gingival phenotype and the underlying bone morphotype (Jepsen et al., 2018). Different techniques have been proposed to assess the gingival phenotype including transgingival piercing with an endodontic reamer, a special ultrasonic device or insertion of a periodontal probe or color‐coded probe into the gingival sulcus (Eger et al., 1996) (Kloukos et al., 2018) (Fischer et al., 2018).

Olsson and Lindhe (1991) found distinctive gingival properties based on tooth shape and gingival thickness: while subjects with a thick biotype showed higher probing depths (DP), the group with a thin biotype were prone to more recessions and, hence, these groups indicated clearly different reaction patterns towards gingival/periodontal disease. The clinical evaluation of soft and hard tissue characteristics as well as crown shape proportions revealed the following main findings: (i) similar buccal and interproximal bone sounding levels (Fischer et al., 2014), (ii) comparable crown shape dimensions with the exception of crown length (Fischer et al., 2016), and (iii) significant different gingival thicknesses when artificially creating “very thin” and “very thick” gingival phenotype subgroups in healthy Caucasian subjects (Fischer et al., 2015). For this reason, a periodontal phenotype probe has been introduced to differentiate between thin, moderate, and thick soft tissues (Fischer et al., 2018).

From a clinical perspective, the diagnostic assessment of the soft tissue dimensions may have a substantial and multi‐disciplinary impact in the decision‐making process for orthodontic, prosthetic, periodontal, and implant treatment (Malpartida‐Carrillo et al., 2021). Already in the early 90s, Wennstrom (1990) highlighted the importance of gingival thickness rather than gingival width with respect to the development of gingival recessions during orthodontic treatment. In plastic periodontal surgery, a positive correlation between flap thickness and complete root coverage was also reported (Hwang & Wang, 2006), indicating, that a successful plastic periodontal surgery might be easier to perform in patients with a thick biotype. In addition, the influence of four different phenotype categories was also recently investigated. The authors found that the very thick and thick phenotypes resulted in superior clinical and esthetic results and that a tunneling approach might be even feasible without a connective tissue graft (CTG) under these circumstances (Rasperini et al., 2019). A very recent network meta‐analysis investigated the possibility of phenotype modification around teeth and reported that especially autogenous soft tissue grafts proved to be able to enhance gingival thickness (Barootchi et al., 2020). Furthermore, greater gingival recession can be seen in thin GB after guided‐tissue‐regeneration (GTR) in furcation defects, immediate implant placement and subgingival crown preparation (Anderegg et al., 1995; Kan et al., 2011; Parma‐Benfenali et al., 1985).

Based on the existing role of the gingival phenotype in many practical aspects, we aimed to screen the gingival biotype at index teeth (so‐called “Ramfjord teeth”: 16, 21, 24, 36, 41, and 44) and to investigate potential differences between women and man. We hypothesized that there is a general GP within one dentition and that index teeth may serve as a full‐mouth screening option.

## 2 MATERIAL AND METHODS

### 2.1 Study design

In this cross‐sectional observational study, dental students were randomly screened on a voluntary basis and the distribution of thin and thick gingival phenotype appearance within one dentition in selected teeth (“Ramfjord teeth”) was assessed.

### 2.2 Participants

The Witten/Herdecke University's Ethical Committee of the Medical Faculty approved the consent form and study protocol (34/2015). Sixty‐four dental students out of the clinical years (3rd to 5th year) of the Witten/Herdecke University were screened for eligibility. The following exclusion criteria were applied:

- Pregnant female volunteers,
- Subjects with cervical fillings or crowns
- Tooth crowding and misalignment,
- Intake of any medication affecting the soft tissue health (e.g., Amlodipine, Cyclosporine A, and Hydantoin),
- Volunteers with either signs of periodontal disease defined as periodontal probing depths >3 mm or gingival recessions,
- Heavy smokers (>10 cigarettes/day).

Each participant signed informed consent form after thorough explanation of the nature, risks, and benefit of this clinical investigation.

### 2.3 Interventions before evaluation

All subjects received oral hygiene instructions and, if needed, and cleaning and polishing of all teeth.

After calibration using digital photographs, one dentist (KF) obtained all clinical parameters. Five test‐subjects were examined prior starting with enrolment and data collection.

### 2.4 Outcome and clinical parameters

Primary outcome measure: To assess the gingival phenotype based in probe transparency through the gingival margin within on dentition measured on index teeth (“Ramfjord”‐teeth: 16, 21, 24, 36, 41, and 44).

Secondary outcome measures: connection between the gingival phenotype, tooth position in view of the individual gender.

**Figure 1:**
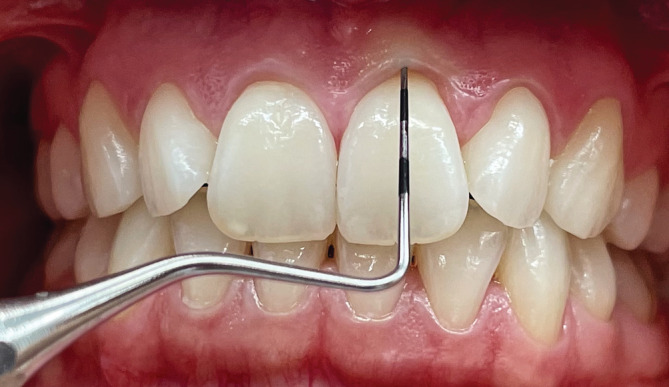
Clinical evaluation of GP of a male participant based on probe transparency through the gingival margin; probe tip as not visible at tooth 21, hence, GP is categorized as thick

Gingival phenotypes (GP) were dichotomously categorized as thin or thick based on the visible transparency of a periodontal probe at teeth 16, 21, 24, 36, 41, and 44 (PCP12, Deppeler SA, Rolle, Switzerland) (Kan et al., 2003). Transparency was evaluated through the gingival margin while probing the sulcus at the mid‐facial aspect as described earlier (Kan et al., 2010) or in simple words: In cases where the probe was not visible through the tissue, the gingival biotype was categorized as “thick.” In contrast, if the probe tip could be easily identified through the sulcus, the gingiva was classified as “thin” (Figure 1(a) and (b)).

In addition, the following clinical parameters were measured around tooth 21 as recently published Fischer et al. (2018):

1. Mid‐facial probing depth (PD) was measured to the nearest 0.5 mm with a millimeter‐scaled pressure controlled periodontal probe (EZ Probe, Natural Law, Seoul, Korea),
2. Gingival thickness (GT) was measured at tooth 21 using a customized digital caliper with a minimal spring force of 4 g/mm^2^. GT was determined before phenotype assessment to avoid any bias.
3. Gingival width (GW) was evaluated mid‐buccally to the nearest of 0.5 mm with a periodontal probe at tooth 21. GW was defined as the distance between the free gingival margin and the mucogingival junction (De Rouck et al., 2009). In case the mucogingival junction was not clearly detectable, the “wrinkle‐test” was used (Olsson et al., 1993).

### 2.5 Statistical analysis

Fisher's Exact Tests were performed to compare female versus male participants, upper versus lower jaw per tooth location. Significance level was set at *α* = 0.05. Bonferroni‐correction was applied for multiple testing.

## 3 RESULTS

### 3.1 Baseline data

After screening, four volunteers dropped out because of restorations at tooth 21 (Fischer et al., 2018) and another four subjects had to be excluded because of extensive restorations at the molars. Finally, 56 volunteers (all Caucasians; 39 females and 21 males) could be enrolled in this study with an age ranging from 19 to 37 (mean: 23). Mean mid‐facial PD_21_ was 2.2 mm without reaching a statistically significant difference between thin and thick phenotypes (*p* > 0.05). Mean GT_21_ was 0.6 mm (SD: 0.17 mm) and mean GW21 was 4.73 mm (SD: 1.25). Furthermore, GT_21_ and GW_21_ appeared to be significantly directly correlated (Spearman correlation: *p* < 0.001; *R* ^2^: 0.308).

### 3.2 GP distribution

Only seven subjects presented with a constant gingival phenotype over all “Ramfjord” index teeth: Five participants (male: three/female: two) displayed a uniform and constantly thick GP, whereas two female participants had a constantly thin GP. No male participant showed a uniformly thin phenotype.

Regarding phenotype distribution in the upper and lower jaw, more maxillary teeth showed a thick phenotype (59%), whereas in the lower jaw, the distribution was almost equal with 49.4% thick and 50.6% thin phenotypes, respectively (*p* = 0.001).

**Figure 2:**
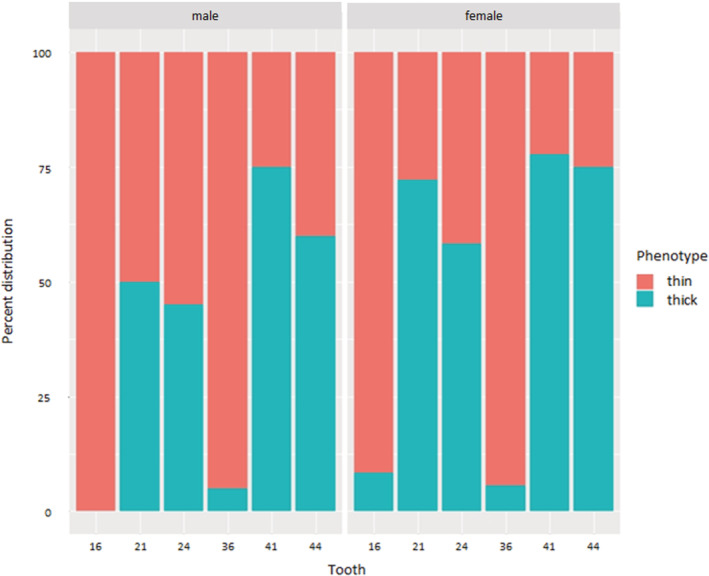
Comparison of GP distribution (in %) between female and male participants assessed at the “Ramfjord” index teeth; while 94.6% of molars were categorized into thick GP, 70.5% of incisors and 61.6% of premolars were found to show a thin GP, respectively

The clear majority of molars (94.6%) were categorized as thick with a highly significant difference (*p* = 0.006). In contrast, 61.6% of premolars (24: 51.7%/44: 67.5%) and 70.5% of incisors (21:61.1%/41:76.8%) were categorized as thin. While no statically significant difference was found for the premolar area (*p* = 0.09), slightly more thin GP were found for the incisor area (*p* = 0.046) (Figure 2).

Regarding the differences between female versus male participants, no statistical significant difference was found, neither in general (*p* = 0.722) nor with respect to tooth side (16: *p* = 0.55/21: *p* = 0.15/24: *p* = 0.41/36: *p* = 1.00/41: *p* = 0.53/44: *p* = 0.36).

## 4 DISCUSSION

In this investigation, we sought to assess the relation between clinically estimated gingival phenotype and its distribution within one dentition using the “Ramfjord” index teeth and compared the outcomes of women and men. To the authors best knowledge, this is the first clinical investigation reporting on phenotype distribution in the upper and lower jaw as well as in molars, premolars and incisors, respectively – also with regard to gender differences.

Based on our findings, the hypotheses of a uniform GP within one appears very questionable. Constant GP was found in only five out of 56 participants. While the clear majority of molars were categorized as having a thick GP, most of incisors and premolars were found to have a thin GP, respectively. More maxillary teeth seem to have a thick GP and, in contrast, a nearly equal distribution was reported for the mandible. Regarding potential differences between female and male participants, no statistical significant difference was found. Nevertheless, comparing the percentages of thick versus thin GP in relation to gender, a tendency for more widespread thick GP can be observed for male participants (Figure 2).

The clinical assessment of gingival biotypes using a periodontal probe was introduced as an easy and low‐cost technique by Kan et al. (2003) in all day clinical practice. Later, the same group (2010) compared this indirect assessment with a visual estimation and a direct measurement after tooth extraction. In fact, the visual evaluation was found to be unreliable, while the use of a probe showed no statistical significant difference to the direct measurement and may therefore be regarded as an objective clinical method. This is in accordance with Eghbali et al. (2009) showing the difficulties of correctly assessing gingival assessment independent of the examiners experience. To date, mostly upper anterior teeth have been investigated in relation to different GP focusing mainly on dental implant treatment (Cosyn et al., 2013, 2016; Kao et al., 2008; Lee et al., 2011; Nisapakultorn et al., 2010) and showing less mucosal recession for thick GP. Earlier reports, however, also focused on GT in relation to tooth type and position but only reporting on either a male (Müller & Eger, 1997) or female (Muller & Kononen, 2005) population without differentiation into different GP. These reports found clear correlations between tooth characteristics as well as tooth location and GT as seen in our study for GP. Furthermore, palatal masticatory mucosa seems to depend on GP and gender with thinner mucosa for women compared to men (Muller et al., 2000). Assessing GT in anterior teeth in an Indian population of varying age and equally distributed gender groups, younger age, male gender, and tooth location in the maxilla was associated with thicker gingiva (Vandana & Savitha, 2005). While we did not assess different age groups, we also found a tendency for a more frequent thick GP in the upper jaw and slightly more frequent thick GP for young men.

Limitations of this report might be the relative small sample size and the sole application of a single assessment method based on probe transparency by only one clinician without testing for intra‐rater repeatability. Inter‐rater reliability could not be tested because of the study design. Past orthodontic treatment, furthermore, was not considered as potentially soft tissue thickness influencing factors. All participants were asked about orthodontic therapies and nearly 90% remembered such a treatment. Hardly any participant, however, could report on applied techniques or movements and, hence, further evaluation was discarded. In addition, crowding and tooth angulation was appraised only clinically, hence might be seen as not objective. Furthermore, we did not evaluate our study population in a case–control manner, and, therefore, this also might be a limitation. Future research might include comparison of gingival configuration around dental implants and natural teeth or around natural teeth before and after orthodontic treatment. To establish a screening protocol based on index teeth, screening of the whole dentition or at least contralateral teeth with regard to GP, GT, and GW might be advisable.

In conclusion, diverse gingival thickness must be expected within one dentition with a clear differentiation based on tooth location. An actual screening based on index teeth seems therefore feasible. Molars, in general, have thicker soft tissues than premolars and incisors, respectively, and there seems to be a clear tendency for a thicker phenotype in the maxilla compared to the mandible as well as slight tendency in young men compared to women for maxillary teeth again. Interventional studies need to evaluate the influence of these findings for different dental treatments with larger subject numbers.

## CONFLICT OF INTEREST

The authors declare that they have no conflict of interest.

## AUTHOR CONTRIBUTIONS

Kai Fischer, Anton Friedmann, and Patrick Schmidlin contributed to the study conception and design. Material preparation, data collection, and analysis were performed by Jasmin Büchel, Kai Fischer, Frederic Kauffmann, and Christian Heumann. The first draft of the manuscript was written by Kai Fischer and all authors commented on previous versions of the manuscript. All authors read and approved the final manuscript.

## Data Availability

The data that support the findings of this study are available from the corresponding author upon reasonable request.
